# Progress in Psychiatry

**Published:** 1900-09-08

**Authors:** 


### PROGRESS IN PSYCHIATRY.

(*Continued from page* 371.)

**Toxic Action of Alcohol.**—Having regard to the conditions attending the operation of toxic agents as just alluded to, it may be said that alcohol stands foremost (after heredity) as a distinct and potent cause of insanity. The most recent statement on its value as an ætiological factor makes it account for 25 per cent. of all cases admitted to lunatic asylums,[50] the proportion of cases of the male sex exceeding that of the female sex in the proportion of three or four males to one female. The effects of alcohol may be manifested in various forms clinically, *e.g.*: (*a*) Acute alcoholic intoxication, with a general paralysis of thought, intelligence, and voluntary movement tending to coma[51]; (*b*) alcoholic delirium tremens; (*c*) alcoholic mania à potu; (*d*) chronic alcoholic insanity with or without polyneuritis; and (*e*) psychosis polyneuritica. The visceral diseases accompanying chronic alcoholism are gastric catarrh, cirrhosis of the liver preceded by a congestive-fatty (nutmeg) condition, chronic nephritis combined with more or less fatty degeneration, fatty heart, atheroma and arterio-sclerosis. Polyneuritis, with tenderness of muscles and nerve-trunks in the legs and arms, local muscular paresis and wasting, paræsthesiæ and neuralgias are local manifestations. The peculiar cerebral disease resulting from chronic alcoholism is very characteristic, and will be referred to later in its proper place. The acute and sub-acute manifestations take the three forms (*a*, *b*, *c*) above noted. Usually acute alcoholic intoxication or drunkenness is marked by the peculiar character of the mental condition which we call "elevated," and by the clinical signs accompanying it, such as thickness and blurring of speech, reeling and unsteady gait, clumsiness and awkwardness of movement of hands and fingers. These manifestations resemble to some extent those of moderately-advanced general paralysis of the insane. When the drunken man is a man of ordinarily strong and sound constitution the manifestations of drunkenness take the above ordinary form. But when he is a neurotic, with unbalanced disposition of the brain, the manifestations differ accordingly. In such a case the disorders of articulation and of gait may take on quite another form, and "the total disorder of conduct may amount to a violent outbreak of maniacal delirium; so that the drunkard, instead of reeling home in a state of maudlin besottedness, raves, screams, smashes the furniture, strips himself naked, jumps through the window, murders or tries to murder an inoffensive bystander, and is taken to a lunatic asylum as a dangerous maniac, which he is."[52] Such cases, if deficient in self-control and determination, cannot keep from the bottle, and pass their lives going in and out of asylums. In other cases the drunkard remains sullen, morose, suspicious, and revengeful. In others, again, he becomes the ready subject of terrifying hallucinations or delusions, and if these assume an acute stage, and there are present bodily weakness and tremor, the combination of symptoms constitutes delirium tremens. The habitual drunkard whose disease stops short of insanity is prone to illusions and hallucinations, especially of sight. A tendency to nocturnal delirium frequently prevails, combined with a considerable degree of amnesia for recent events. Persons and their names are very readily forgotten. The patient's waking state is a continuation of his dream-vigils and nocturnal semi-delirium. With these psychical symptoms, to which must be added a rather frequent tendency to suicide, are combined the somatic symptoms of alcoholism, such as diffused tremors, cramp, and paræsthesiæ of the limbs, dyspeptic disorders, epileptiform and hysteriform convulsions—the whole taking a sub-acute course. If the patient is taken under care, and abstinence is enforced, the dream-like delirious conditions and troubles disappear after the first week, and a restoration to normal waking life soon begins to be noticeable, though for several days longer hypnagogic hallucinations may persist. Just as insomnia marks the beginning of the disease, so the return of sleep marks its close.

Many authors, notably Lancereaux (1891)[53], have tried to differentiate alcoholism according to the nature of the drink indulged in. Some of the distinctions are important, and consist in the great frequency of epileptiform and convulsive accidents resulting from absinthe drinking, the hyperalgesia resulting from intoxication with liquors and essences, and other minor differences. Hereditary degenerates suffer with great facility from the mental troubles and cerebral accidents of alcoholism, and these have been described at considerable length by Magnan and Legraine.[54]

Chronic alcoholism, the result of heavy drinking indulged in for many years, is marked by a characteristic group of symptoms which have been carefully studied and classified by Lloyd Andriezen[55] under the following heads :—

First, diminished power of recollection (amnesia), *i.e.*, of revivifying past mental image or ideas.

Second, diminished power of attention and volition.

Third, diminished initiativeness and energy in conduct.

Fourth, diminished muscular power, and trembling (neurasthenia).

Fifth, blunting of higher moral and ethical sense.

Sixth, insomnia, loss of capacity for sleep and recuperation.

Seventh, serious disturbances in the balance of cortical representations of external world and empirical ego, with melancholia and suspicion, delusions of persecutions, excitement, hallucinations, gloomy feelings and pathetic emotional state, and suicidal tendencies.

In patients with a comparatively healthy heredity, the above symptoms are gradually developed, and a slow and progressive mental enfeeblement ensues which passes into a state of dementia. In subjects of a neurotic heritage, or in those allied to the epileptic, the criminaloid and the paranoiac temperament, the group of symptoms above mentioned is "rapidly passed over; and the special and intense insane involvement makes its appearance early, viz., as melancholia, with persecutory delusions (two-thirds of the total number of cases) with or without ideas of grandeur, varied by outbursts of acute excitement (mania), in which acute hallucinations play at times a causal part. These hallucinations and delusions may be aural, visual, olfactory, cutaneous, gastric and abdominal, genito-urinary, &c., or several of these combined, and having affinities with the more rapidly evolving general paralytic on the one hand, and the more slowly developing paranoiac on the other."[56] Many cases of chronic alcoholism have symptoms like those of general paralysis, and the differential diagnosis is often difficult (alcoholic pseudo-paralysis).[57] Alcoholic pseudo-general paralysis is found only in well-marked cases of chronic alcoholism. It begins in some cases with apoplectic form or epileptiform attacks, which differ in certain respects from those of general paralysis. At other times (and this more frequently) the pseudo-general paralysis is consecutive to a sub-acute attack of alcoholism. In all cases, instead of being insensibly progressive, like true general paralysis, the symptoms of pseudo-general paralysis at once attain their greatest intensity. Further, focal paralyses, such as permanent monoplegias, hemiplegias, and aphasias are more frequent and more persistent than in true general paralysis. Pseudo-general paralysis habitually ends in recovery, and may be reproduced repeatedly after new excesses. Ball and Regis have recorded the case of a patient who recovered 16 times in 13 years from alcoholic pseudo-general paralysis. Insanity from morphia presents the following characters. The earliest symptom is a general sensation of disquiet, manifested by incoherence of thought, difficulty of concentration of the mind, marked restlessness and insomnia. The chief physical disorders are anorexia and constipation, anæmia, cardiac weakness and intermittence, and bradycardia, miosis or pin-point contraction of the pupils in the early stages, and mydriasis later, with sluggishly-reacting pupils, impotence in men, and amenorrhœa in women; headaches and localised shooting pains, including neuralgias and paræsthesiæ; frequent loss of knee-jerk, diminished sensibility to touch and pain, and concentric limitation of the visual field. Elementary sensorial disturbances such as tinnitus and muscæ volitantes, loss of volitional power, moral perversion, with fair retention of memory, and a proneness to intellectual fatigue are the main cerebral symptoms. A well-marked attack of insanity in a subject addicted to the morphia habit is more often the result of enforced abstinence than of chronic misuse of the drug, and the mental excitement and agitation may resemble that of acute mania.

[50] Dr. Percy Smith's Presidential Address at the Psychology section at the meeting of the British Medical Association, "On the Prevention of In-anity," Brit. Med. Jour., Aug. 11. [51] An excellent account is given by Dr. Charles Mercier in his Sanity and Insanity, p.314-21. [52] Ibid., p. 315. [53] Bulletin Medical, 1891. [54] Legraine, Heredite et Alcoolisme, Paris. [55] On some of the Newer Aspects of the Pathology of Insanity, Brain, Winter, 1894, p. 665-687. [56] Ibid., p. 686. [57] Regis, Mental Medicine, p. 994-503.

